# A case of descending colon cancer achieving a complete response with preoperative capecitabine plus oxaliplatin therapy following colorectal stenting

**DOI:** 10.1093/jscr/rjag101

**Published:** 2026-02-26

**Authors:** Chikai Mitsuhara, Masayoshi Nishihara, Naoya Ozawa, Shima Asano

**Affiliations:** Department of Emergency and Critical Care Medicine, Osaka Keisatsu Hospital, 2-6-40 Karasugatsuji, Tennoji, Osaka 543-0042, Japan; Department of General Surgery, Okinawa Miyako Hospital, 427-1 Shimozato, Hirara, Miyakojima, Okinawa 906-0013, Japan; Department of General Surgery, Okinawa Miyako Hospital, 427-1 Shimozato, Hirara, Miyakojima, Okinawa 906-0013, Japan; Department of General Surgery, Okinawa Miyako Hospital, 427-1 Shimozato, Hirara, Miyakojima, Okinawa 906-0013, Japan; Department of General Surgery, Okinawa Miyako Hospital, 427-1 Shimozato, Hirara, Miyakojima, Okinawa 906-0013, Japan

**Keywords:** obstructive colon cancer, colorectal stent, complete response, neoadjuvant chemotherapy, CapeOX

## Abstract

A 71-year-old woman with obstructive descending colon cancer and synchronous lymph node and liver metastases underwent colorectal stenting to relieve colonic obstruction. Preoperative capecitabine plus oxaliplatin (CapeOX) was administered for four cycles. Subsequent imaging showed marked shrinkage of the primary tumor, with nodal and hepatic lesions no longer detectable. Laparoscopic left hemicolectomy was then performed. Histopathological examination demonstrated a pathological complete response (CR), with no viable tumor cells in the primary lesion and no lymph node metastasis. Postoperative chemotherapy was resumed, and the patient remains recurrence-free. This rare case highlights a CR in both primary and metastatic lesions after colorectal stenting and preoperative CapeOX therapy.

## Introduction

In recent years, neoadjuvant chemotherapy (NAC) has received attention for colorectal, esophageal, pancreatic, and breast cancers. We report a case of descending colon cancer with liver metastases treated with preoperative capecitabine plus oxaliplatin (CapeOX) therapy following colorectal stenting, and achieved a pathological complete response (pCR).

## Case report

A 71-year-old woman was referred for further evaluation after anemia (hemoglobin 5.8 g/dL) was incidentally detected. Her medical history included cataracts, asymptomatic hepatitis B virus carrier status, and *Helicobacter pylori* infection. On referral, her hemoglobin was 8.2 g/dL, and tumor markers were elevated (CEA, 71.9 ng/mL; CA19–9, 87.2 U/mL).

### Lower gastrointestinal endoscopy

**Figure 1:**
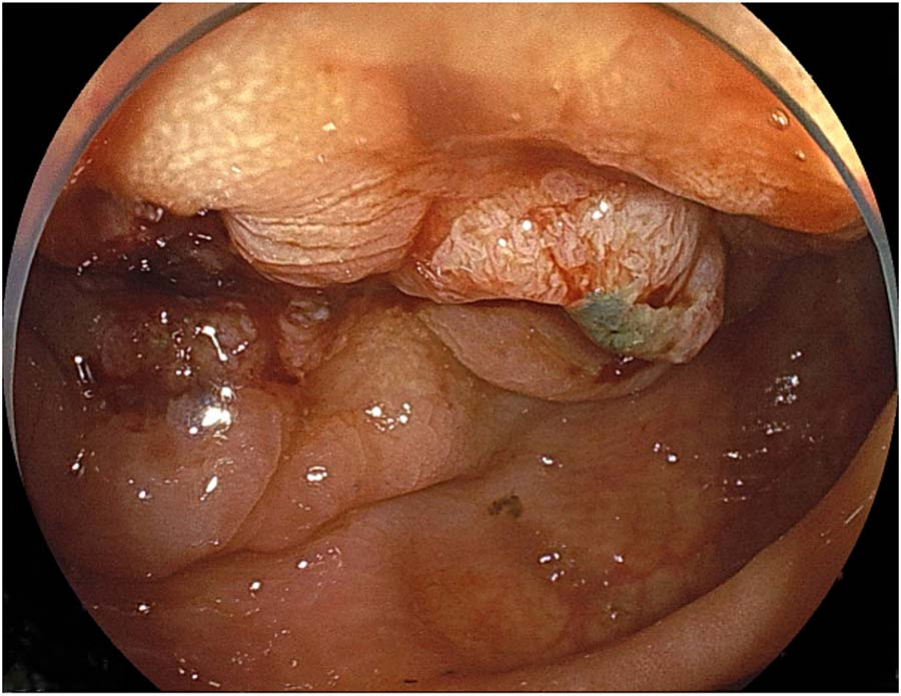
Circumferential type II tumor with severe stenosis in the descending colon.

Endoscopy demonstrated a circumferential type II tumor with severe stenosis in the descending colon (Fig. 1). A self-expandable metallic stent (Boston Scientific HANAROSTENT Naturfit, 22 × 8 cm) was placed to relieve the obstruction.

### Computed tomography

**Figure 2:**
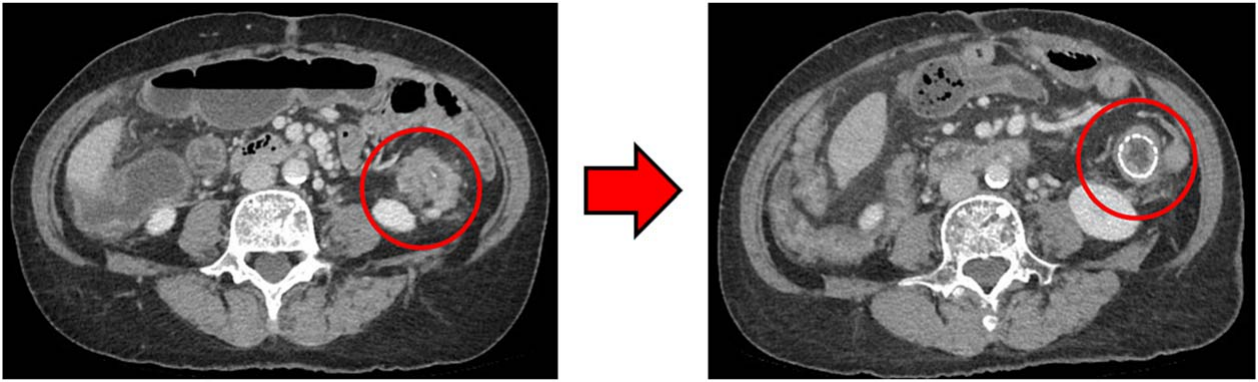
(Left: Before NAC) Wall thickening of the descending colon with increased pericolic fat tissue density. (Right: After NAC) Significant shrinkage of the primary lesion.

**Figure 3:**
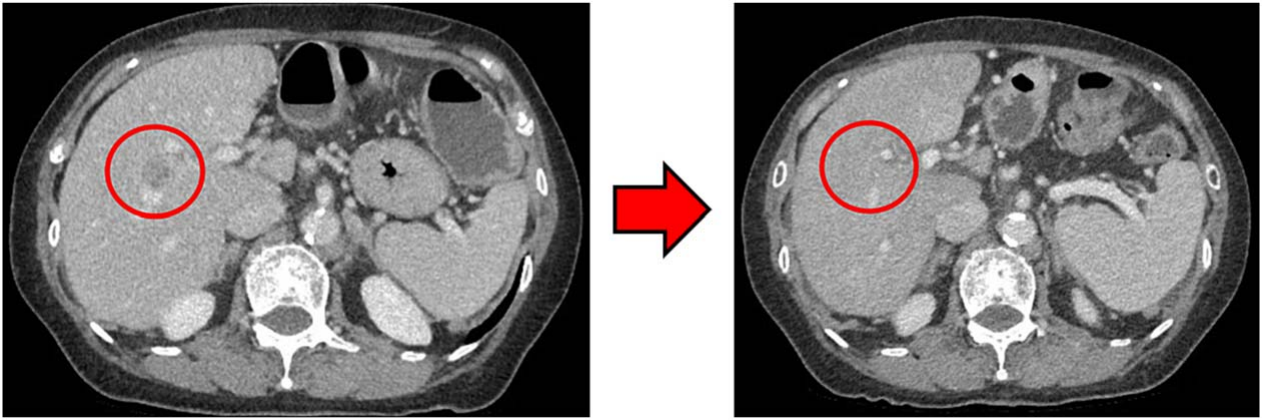
(Left: Before NAC) Liver metastasis in the liver S5/7. It was located near the anterior branch of the portal vein and the right hepatic vein. (Right: After NAC) Liver metastasis in the liver S5/7. Complete resolution of the S5/7 liver metastasis.

**Figure 4:**
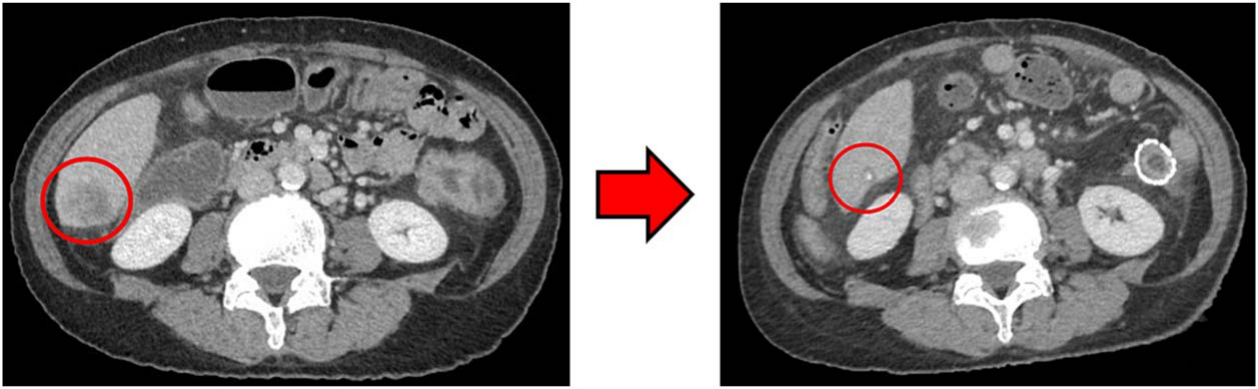
(Left: Before NAC) Liver metastasis in the liver S6. (Right: After NAC) Liver metastasis in the liver S6. It had decreased substantially, with near-complete necrosis.

Computed tomography (CT) revealed wall thickening of the descending colon with increased pericolic fat tissue density (Fig. 2). Multiple enlarged regional lymph nodes were observed. Liver metastases were identified in the liver S5/7 and S6 (Figs 3 and 4). The S5/7 lesion was located near the anterior branch of the portal vein and the right hepatic vein.

### Pathology

Biopsy confirmed adenocarcinoma (tub1 > tub2) with RAS mutation, BRAF wild-type, and microsatellite instability-negative status.

### Clinical course

The patient was diagnosed with cT4a N2b M1a, Stage IVa. Considering the synchronous liver metastasis and risk of anastomotic leakage, NAC was selected. CapeOX was initiated (oxaliplatin 195 mg on day 1; capecitabine 3000 mg/day on Days 1–14).

After four cycles, CT revealed significant shrinkage of the primary lesion (Fig. 2), disappearance of lymph node metastases, and complete resolution of the S5/7 liver metastasis (Fig. 3). The S6 lesion had decreased substantially, with near-complete necrosis (Fig. 4). Tumor markers declined (CEA 16.7 ng/mL, CA19–9 31.1 U/mL).

Curative resection was deemed feasible, and laparoscopic left hemicolectomy with partial hepatectomy was planned.

### Surgical findings

Intraoperative ultrasound detected no residual liver metastases. Thus, only laparoscopic left hemicolectomy with D2 lymphadenectomy was performed. An additional proximal resection was required due to poor blood flow. The operation lasted 265 minutes, with an estimated blood loss of 50 mL.

### Pathology

**Figure 5:**
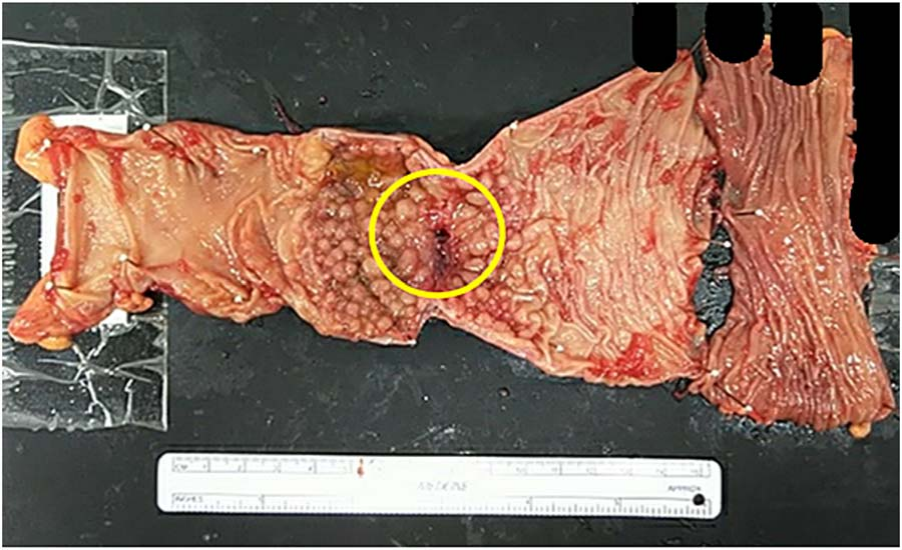
Resected specimen. Scar tissue corresponding to the primary site was identified in the resected specimen.

**Figure 6:**
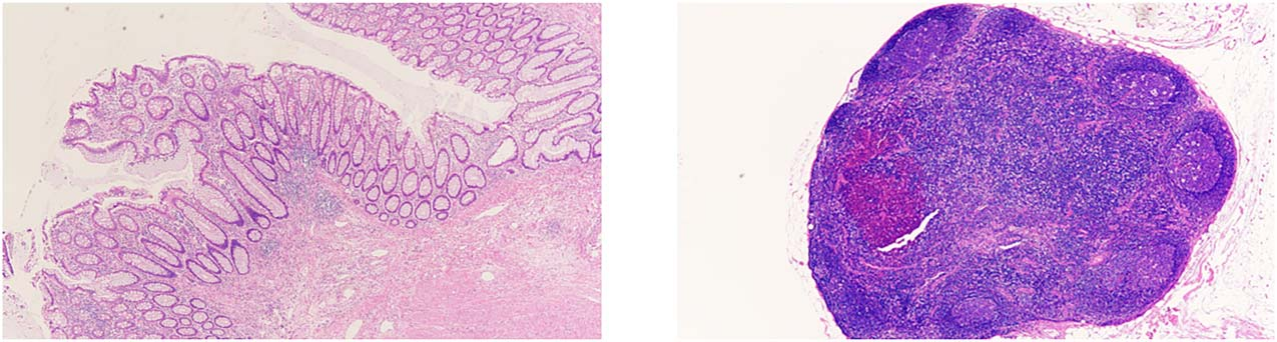
(Left) Primary site. No residual tumor cells were observed. (Right) Lymph nodes. No lymph node metastases were found.

Scar tissue corresponding to the primary site was identified in the resected specimen (Fig. 5). No residual tumor cells at the primary site, and no lymph node metastases were observed [ypT0, ypN0 (0/26)] (Fig. 6). The histological evaluation of the therapeutic effect was grade 3.

### Postoperative course

The patient was discharged on postoperative Day 23.

Liver metastases were considered in remission, CapeOX chemotherapy was resumed, and the patient remains recurrence-free.

## Discussion

Approximately 10%–15% of patients with advanced colorectal cancers present with obstruction [1]. Since 2012, colorectal stenting has been covered by insurance in Japan as palliative treatment and as a bridge to surgery (BTS) [2]. Initially, the European Society of Gastrointestinal Endoscopy (ESGE) issued negative recommendations regarding BTS due to long-term concerns [3]. However, multiple comparative studies reported no significant differences in survival between BTS and emergency surgery [4–6], leading ESGE to revise its recommendations in 2020 [7]. Nonetheless, numerous issues remain to be resolved, including recurrence rates [8–10] and optimal surgical timing [11, 12].

Considering these problems, our institution has adopted NAC after stenting, with patient consent, since July 2023. The goal is to reduce anastomotic leakage caused by obstructive colitis and to improve R0 resection rates. CapeOX was chosen because of its immediate implementability and high tolerability. Bevacizumab is avoided due to the risks of bleeding, perforation, and anastomotic leakage. After 3–6 cycles, CT and lower gastrointestinal endoscopy are repeated to reassess tumor status and detect proximal lesions. In this case, curative resection was judged to be feasible after four cycles, so surgery was performed 120 days after stenting.

The optimal interval between stenting and surgery is unclear. Longer intervals may reduce postoperative complications, including anastomotic leakage [11]. Conversely, shorter intervals may decrease stent-related complications and tumor recurrence [12]. Furthermore, stent-induced tissue changes influence staging accuracy. When NAC is introduced, interval determination becomes even more complex.

In Japan, surgery followed by adjuvant chemotherapy remains standard for locally advanced or resectable stage IV colorectal cancer. In contrast, in Western countries, neo-chemoradiotherapy (nCRT) for rectal cancer and nonoperative management after clinical complete response (CR) achieved by nCRT is becoming mainstream. Recently, the significant tumor-shrinking effects of chemotherapy for colorectal cancer have drawn attention to the use of NAC for advanced colon cancer. In the pilot phase of the FOxTROT trial, the NAC group (FOLFOX) showed significantly better R0 resection rates and notable pathological tumor shrinkage compared with the surgery-first group [13]. The phase III FOxTROT trial showed significantly better R0 resection rates and a trend toward lower two-year recurrence rates in the NAC group [14]. In a study by Huabin Hu, patients with locally advanced T3/T4 colon cancer assigned to NAC (mFOLFOX6 or CapeOX) had similar three-year disease-free survival rates compared with surgery-first; however, 7% of NAC patients achieved a pCR, with reduced depth of invasion and lymph node metastasis [15].

Discussions on the introduction of NAC for advanced colon cancer are beginning in Japan, and this case may serve as a valuable reference for advancing these discussions.

To the best of our knowledge, there are limited reports of obstructive colon cancer managed with colorectal stenting followed by neoadjuvant CapeOX without molecular-targeted agents, resulting in a pCR.

## Conclusion

This case represents a rare instance in which a CR in both primary and metastatic lesions of descending colon cancer (cStage IVa) was achieved with preoperative CapeOX therapy following colorectal stenting. This experience supports our institutional NAC protocol and may provide a useful reference for the design of future large-scale clinical trials.

## Data Availability

The datasets used during this case report are available from the corresponding author upon reasonable request.

## Presentation at a conference

We presented this case at the 80th General Meeting of the Japanese Society of Gastroenterological Surgery, held from 16 July to 18 July 2025 at the Kobe Convention Center.
